# Treatment of cardiac arrhythmias in a mouse model of Rett syndrome with Na^+^-channel-blocking antiepileptic drugs

**DOI:** 10.1242/dmm.020131

**Published:** 2015-02-20

**Authors:** José A. Herrera, Christopher S. Ward, Meagan R. Pitcher, Alan K. Percy, Steven Skinner, Walter E. Kaufmann, Daniel G. Glaze, Xander H. T. Wehrens, Jeffrey L. Neul

**Affiliations:** 1 Interdepartmental Program in Translational Biology and Molecular Medicine, Baylor College of Medicine, Houston, TX 77030, USA.; 2 Jan and Duncan Neurological Research Institute, Texas Children’s Hospital, Houston, TX, USA.; 3 Department of Pediatrics, University of Alabama at Birmingham, Birmingham, AL, USA.; 4 Greenwood Genetics Center, Greenwood, SC 29646, USA.; 5 Department of Neurology, Boston Children’s Hospital, Boston, MA 02115, USA.; 6 Department of Pediatrics, Baylor College of Medicine, Houston, TX 77030, USA.; 7 Cardiovascular Research Institute, Department of Molecular Physiology and Biophysics, Baylor College of Medicine, Houston, TX 77030, USA.

**Keywords:** Long QT, Rett syndrome, Propranolol, Phenytoin, Arrhythmia, MECP2

## Abstract

One quarter of deaths associated with Rett syndrome (RTT), an X-linked neurodevelopmental disorder, are sudden and unexpected. RTT is associated with prolonged QTc interval (LQT), and LQT-associated cardiac arrhythmias are a potential cause of unexpected death. The standard of care for LQT in RTT is treatment with β-adrenergic antagonists; however, recent work indicates that acute treatment of mice with RTT with a β-antagonist, propranolol, does not prevent lethal arrhythmias. In contrast, acute treatment with the Na^+^ channel blocker phenytoin prevented arrhythmias. Chronic dosing of propranolol may be required for efficacy; therefore, we tested the efficacy of chronic treatment with either propranolol or phenytoin on RTT mice. Phenytoin completely abolished arrhythmias, whereas propranolol showed no benefit. Surprisingly, phenytoin also normalized weight and activity, but worsened breathing patterns. To explore the role of Na^+^ channel blockers on QT in people with RTT, we performed a retrospective analysis of QT status before and after Na^+^ channel blocker antiepileptic therapies. Individuals with RTT and LQT significantly improved their QT interval status after being started on Na^+^ channel blocker antiepileptic therapies. Thus, Na^+^ channel blockers should be considered for the clinical management of LQT in individuals with RTT.

## INTRODUCTION

Rett syndrome is an X-linked dominant neurodevelopmental disorder that primarily affects females and has an incidence of one in 10,000 female births (Hagberg, 1985). Mutations in Methyl-CpG-binding protein 2 (*MECP2*), a transcriptional regulator (Chahrour et al., 2008), cause the majority of RTT cases (Amir et al., 1999). RTT is characterized by loss of spoken language, loss of hand skills, abnormal gait and repetitive purposeless hand stereotypies. A number of additional clinical features are prominent including seizures (Glaze et al., 2010), breathing abnormalities (Neul et al., 2010), autonomic dysfunction (Julu et al., 2001), and prolonged (long) QT (LQT) intervals (McCauley et al., 2011; Sekul et al., 1994). Additionally, nearly a quarter of deaths in RTT are sudden and unexpected, and it is suspected that prolongation of the QT intervals might lead to sudden cardiac death in some of these unexpected deaths (Ellaway et al., 1999; Guideri and Acampa, 2005; Guideri et al., 2001; McCauley et al., 2011; Sekul et al., 1994).

Mouse models of RTT display many of the clinical features seen in RTT, and both male (*Mecp2^Null/Y^*) and female (*Mecp2^Null/+^*) mice recapitulate the LQT observed in people with RTT (McCauley et al., 2011). Furthermore, these mouse models show increased susceptibility to induced ventricular tachycardia (VT) and sudden cardiac death (SCD), supporting the belief that LQT in RTT underlies sudden death (McCauley et al., 2011). The mouse models provide a useful platform to understand the pathophysiology of RTT and to perform pre-clinical testing of potential therapies for the treatment of cardiac abnormalities in RTT.

Recent work has demonstrated that the cardiac phenotypes in *Mecp2^Null/Y^* mice are not responsive to acute therapy with β-adrenergic antagonists (β-blockers such as propranolol) but are responsive to acute treatment with Na^+^ channel blockers such as phenytoin (McCauley et al., 2011), similar to long QT syndrome 3 (LQT3) animal models that have Na^+^ channel abnormalities (Fabritz et al., 2010). This suggests that the optimal treatment of LQT in RTT is through Na^+^ channel blockers. However, because beneficial effects of β-blockade therapy might require chronic-dosing-induced remodeling of cardiac channels, chronic treatment with propranolol might be required for efficacy in RTT mice.

The current standard of care to treat LQT in RTT is through β-blockers, such as propranolol or atenolol; however, the results from mouse studies suggest that this treatment might not be effective. In order to conclusively determine whether this standard of care should continue for this disease, or whether alternative treatment with drugs that block Na^+^ channels should be explored in people with RTT, it is necessary to perform pre-clinical testing in mouse models in order to determine the ability of chronic β-blockade or chronic Na^+^ channel blockade to treat the LQT and prevent VT. Here, we performed a double-blind, randomized, pre-clinical study to test the efficacy of propranolol and of phenytoin in both male and female mouse models of RTT. The primary outcome of the study was arrhythmia prevention; however, drug-dependent physiological and behavioral effects were also monitored. We found that chronic phenytoin, but not chronic propranolol, successfully normalized LQT and prevented induction of VT in both male and female RTT mice. Additionally, phenytoin improved weight and activity in RTT animals, but caused worsening of abnormal breathing patterns in male RTT mice. To further strengthen our hypothesis that antiepileptic drugs (AEDs) with Na^+^-channel-blocking activity are effective in ameliorating the QTc burden, we retrospectively analyzed QTc status pre and post AED therapy from the Rett Natural History Study and found that initiation of Na^+^-channel-blocking AED treatment was associated with normalization of LQT in people with RTT. In total, this work indicates that a new therapeutic approach toward treatment of LQT in RTT needs to be considered.

**TRANSLATIONAL IMPACT**

**Clinical issue**

Rett syndrome (RTT) is a neurological disorder that occurs at an incidence of 1 in 10,000 live female births. There is no cure for RTT, and clinical management and treatment are dependent on the symptoms present in a case-by-case basis. Unfortunately, 25% of all deaths in RTT are sudden and unexpected. 18% of individuals with RTT also present with long QT (LQT), a heart rhythm abnormality associated with a prolongation of the heart QT interval that can lead to sudden death. The standard therapy for those individuals with LQT is treatment with β-blockers. However, acute treatment with the β-antagonist propranolol has been previously shown ineffective in preventing these cardiac problems in RTT mice, suggesting that chronic treatment might be required for efficacy. Additionally, acute treatment with the Na^+^ channel blocker antiepileptic drug phenytoin has proved to be effective in the management of LQT and arrhythmias in RTT mice. However, further studies needed to be conducted to determine whether this drug could be beneficial or detrimental to other phenotypes associated with RTT.

**Results**

Chronic treatment with propranolol in male and female RTT mice was not effective in ameliorating LQT and arrhythmias. By contrast, chronic treatment with phenytoin effectively normalized the QTc (QT corrected for the heart rate) and arrhythmias in both male and female RTT mice. Propranolol unexpectedly worsened the weight phenotype in RTT mice, whereas treatment with phenytoin improved the obesity phenotype in both male and female RTT mice. In addition, phenytoin increased activity levels in male RTT mice but unfortunately worsened their abnormal breathing patterns. Finally, individuals with RTT and LQT showed significantly improved QT intervals after being started on Na^+^ channel blocker antiepileptic therapies.

**Implications and future directions**

These results indicate that propranolol is not an effective therapy for the treatment of LQT and arrhythmias in mouse models of RTT. By contrast, Na^+^ channel blockers are highly effective and the retrospective analysis on individuals with RTT shows that Na^+^ channel blockers might be beneficial in humans. Thus, Na^+^ channel blocker therapies should be considered for the clinical management of LQT in individuals with RTT. However, the exact cause of LQT in RTT is unknown and should be further investigated to identify novel therapeutic approaches for the treatment of LQT in RTT.

## RESULTS

### Chronic β-blockade does not improve electrocardiogram abnormalities or prevent ventricular arrhythmias in mouse models of RTT

Previously, we have demonstrated that acute β-blockade with propranolol does not prevent ventricular arrhythmias in male *Mecp2^Null/Y^* mice (McCauley et al., 2011). To determine whether chronic β-blockade is an effective therapy in preventing ventricular arrhythmias for RTT, we chronically treated male *Mecp2^Null/Y^* mice and wild-type control animals with propranolol (10 mg/kg body weight), or placebo, administered via an osmotic pump for 28 days. Both *Mecp2^Null/Y^* and wild-type controls were treated with the investigator blinded to genotype and treatment in order to avoid potential bias.

**Fig. 1.**
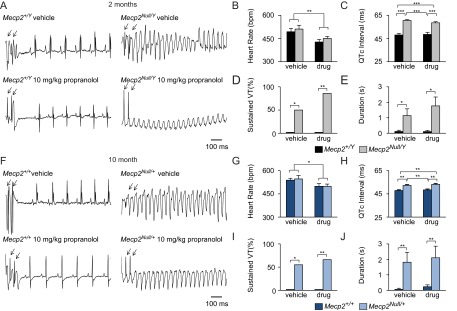
**Chronic β-blockade with propranolol is not an effective therapeutic for arrhythmia prevention in mouse models of Rett syndrome.** (A) Representative traces of normal sinus rhythm after pacing in vehicle- and propranolol-treated *Mecp2^+/Y^* and *Mecp2^Null/Y^* mice. Sustained VT is observed after pacing in both vehicle- and propranolol-treated *Mecp2^Null/Y^* mice. (B) Heart rate data obtained from telemetry in vehicle- and propranolol-treated mice shows the expected decreased heart rate with treatment of β-blockers (*n*=4–5 per genotype per treatment). (C) Quantification of ECG intervals shows a prolonged QTc in both vehicle- and propranolol-treated *Mecp2^Null/Y^* mice. (D) Chronic propranolol is not an effective therapeutic for the prevention of inducible arrhythmias and (E) does not decrease the duration of any arrhythmic event in *Mecp2^Null/Y^* mice. Consistent with the data in males, (F) 10-month-old *Mecp2^Null/+^* vehicle- and propranolol-treated mice both show sustained VT after pacing. (G) β-blockade with propranolol had a treatment effect on heart rate (*n*=4–5 per genotype per treatment), (H) no effect on the prolonged QTc, and (I) is not effective in preventing arrhythmias or (J) decreasing the duration of any arrhythmic in *Mecp2^Null/+^* mice. Data are expressed as means±s.e.m. *n*=7–10 per genotype per treatment unless otherwise stated. *P<0.05, **P<0.01, ***P<0.001. Arrows in A and F show electrical pacing stimulus.

To assess the efficacy of chronic β-blockade to prevent arrhythmia induction in *Mecp2^Null/Y^* mice, we performed *in vivo* right heart catheterization followed by programmed electrical stimulation (PES) and assessed for the induction of sustained VT (Fig. 1A) after pacing stimulus. *Mecp2^Null/Y^* mice were more likely to have VT events after pacing stimulus in both vehicle- and drug-treated mice, regardless of β-blockade treatment (Fig. 1A–E). One of the effects elicited by propranolol is a decrease in heart rate (Epstein et al., 1965; Harrison et al., 1965; Stern and Eisenberg, 1969). To determine whether the propranolol dose was effective, we performed telemetry on a subset of mice prior to PES. Both *Mecp2^Null/Y^* and wild-type propranolol-treated mice exhibited the expected decrease in heart rate (Fig. 1B). Surface ECG measurements and cardiac refractory times were taken prior to PES (supplementary material Table S1). Propranolol had a significant effect on sinoatrial refractory period (SNRT), as previously reported (Kostis et al., 1987), thus providing further evidence of an effective dose (*P*<0.05) (supplementary material Table S1). However, propranolol had no effect on the QTc lengthening observed in *Mecp2^Null/Y^* mice. *Mecp2^Null/Y^* mice presented with a prolonged QTc in both vehicle- and drug-treated *Mecp2^Null/Y^* mice when compared to wild-type control mice (Fig. 1C). Additionally, propranolol failed to prevent arrhythmias in *Mecp2^Null/Y^* mice (Fig. 1D). Finally, *Mecp2^Null/Y^* mice had longer arrhythmic events, including non-sustained and sustained VT, even with propranolol treatment (Fig. 1E).

Given that chronic β-blockade did not prevent VT in male *Mecp2^Null/Y^* mice, we decided to test the female *Mecp2^Null/+^* mouse model of RTT. Although male *Mecp2^Null/Y^* mice recapitulate the phenotypes seen in *Mecp2^Null/+^* at an earlier age (McCauley et al., 2011), the *Mecp2^Null/+^* model is more physiologically relevant to the disorder because affected RTT individuals are nearly always girls and women with heterozygous mutations in *MECP2*. *Mecp2^Null/+^* mice show a progressive onset and worsening of cardiac phenotypes such as the development of LQT and arrhythmias (McCauley et al., 2011) similar to the developmental regression observed in people with RTT (Neul et al., 2014). Even though chronic β-blockade was ineffective in treating cardiac arrhythmias in male *Mecp2^Null/Y^*, it is possible that such treatment would prevent the development of these cardiac problems in female *Mecp2^Null/+^* mice. Therefore, we treated 10-month-old female *Mecp2^Null/+^* and wild-type control animals for 28 days with propranolol (10 mg/kg body weight) via osmotic pump and used PES to assess the ability of this treatment to prevent VT induction and QTc interval prolongation.

Similar to male *Mecp2^Null/Y^* mice, vehicle- and drug-treated *Mecp2^Null/+^* mice were more likely to have VT after pacing stimuli (Fig. 1F). An effective dose of propranolol was provided as demonstrated by the expected decrease in the heart rate (Fig. 1G) and lengthening of the SNRT (supplementary material Table S2). Despite this, propranolol did not shorten the QTc in *Mecp2^Null/+^* (Fig. 1H), or prevent VT induction in *Mecp2^Null/+^* mice (Fig. 1I). Finally, the length in duration of VT in *Mecp2^Null/+^* was not improved with propranolol treatment (Fig. 1J).

### Chronic treatment with the Na^+^ channel blocker phenytoin corrects QTc and abolishes ventricular arrhythmias in mouse models of RTT

Previously, we showed that cardiomyocytes isolated from mice deficient in *Mecp2* have an abnormal Na^+^ current (McCauley et al., 2011) similar to a LQT3 phenotype (Fabritz et al., 2010), and that a single dose of phenytoin prior to PES prevents induction of arrhythmias in male *Mecp2^Null/Y^* mice (McCauley et al., 2011). To determine whether long-term treatment with Na^+^ channel blockers is effective in preventing arrhythmias in RTT mice, we chronically treated *Mecp2^Null/Y^* and wild-type mice with phenytoin (30 mg/kg body weight) or vehicle for 28 days and evaluated the effect on QTc duration and the ability to induce VT using PES.

**Fig. 2.**
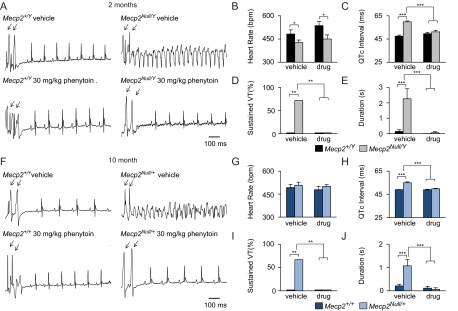
**Chronic phenytoin corrects the prolonged QTc and abolishes sustained VT in male *Mecp2^Null/Y^* and female *Mecp2^Null/+^* mice.** (A) Representative traces of normal sinus rhythm after pacing in vehicle- and phenytoin-treated *Mecp2^+/Y^*, sustained VT in vehicle-treated *Mecp2^Null/Y^*, and normal sinus rhythm after pacing in *Mecp2^Null/Y^* phenytoin treated mice. (B) Heart rate data obtained from telemetry shows no significant effects on heart rate (*n*=4–5 per genotype per treatment). (C) Quantification of ECG intervals shows a prolonged QTc in vehicle-treated *Mecp2^Null/Y^*, which is similar to wild-type levels with phenytoin treatment. (D) Chronic phenytoin abolishes the incidence of sustained VT and decreases the duration of any arrhythmic event in *Mecp2^Null/Y^* to wild-type levels. (E) Consistent with the data in males, 10-month-old *Mecp2^Null/+^* vehicle-treated mice have sustained VT (F) after pacing and *Mecp2^Null/+^* phenytoin-treated mice return to normal sinus rhythm after pacing. Phenytoin treatment did not affect heart rate (*n*=4–5 per genotype per treatment) (G), but normalized the QTc (H), abolished sustained VT (I), and decreased the duration of any arrhythmic event to wild-type levels (J). Data are expressed as means±s.e.m. *n*=7–10 per genotype per treatment unless otherwise stated. *P<0.05, **P<0.01, ***P≤0.001. Arrows in A and F show electrical pacing stimulus.

Interestingly, only vehicle-treated *Mecp2^Null/Y^* mice were susceptible to induced VT after pacing (Fig. 2A). *Mecp2^Null/Y^* mice had a decreased heart rate that was not affected by phenytoin treatment (Fig. 2B). ECG intervals and cardiac refractory periods were quantified to determine the effects of chronic phenytoin treatment (supplementary material Table S3). Phenytoin did not affect refractory periods but did have effects on PR, QRS, and QTc intervals (supplementary material Table S3). Notably, phenytoin treatment corrected the QTc interval of *Mecp2^Null/Y^* mice to wild-type levels (Fig. 2C). Moreover, phenytoin treatment completely abolished VT incidence in *Mecp2^Null/Y^* phenytoin-treated mice (Fig. 2D). Consistent with the PES data, the length of the arrhythmia, including non-sustained VT, was rescued to wild-type values in *Mecp2^Null/Y^* phenytoin-treated mice (Fig. 2E).

Although acute and chronic phenytoin treatments are an effective therapeutic in *Mecp2^Null/Y^* mice, it is unknown whether phenytoin elicits the same effects on female *Mecp2^Null/+^* mice. To further show that Na^+^ channel blockers are beneficial in preventing arrhythmias in mouse models of RTT, we performed pre-clinical experiments on *Mecp2^Null/+^* mice. *Mecp2^Null/+^* mice treated with phenytoin did not present with VT after pacing stimulus whereas *Mecp2^Null/+^* vehicle-treated mice were more likely to have VT (Fig. 2F). Phenytoin did not affect the heart rate of *Mecp2^Null/+^* or wild-type mice (Fig. 2G). To determine the effects of phenytoin, ECG intervals were quantified. Treatment effects were observed on SNRT, atrioventricular refractory period (AVERP), QRS and QTc (supplementary material Table S4). Similar to *Mecp2^Null/Y^* mice, phenytoin rescued the QTc interval to wild-type values (Fig. 2H). Importantly, chronic phenytoin abolished the incidence of VT in *Mecp2^Null/+^* mice (Fig. 2I). Finally, the length of the arrhythmic event was rescued to wild-type values (Fig. 2J).

### Phenytoin increases activity and improves the obesity phenotype of *Mecp2^Null/Y^* mice

Given that Na^+^ channel blockade improved cardiac function in models of RTT, we sought to investigate whether there were other beneficial (or detrimental) effects on the behavior and physiological abnormalities previously reported in these animals (Pitcher et al., 2013; Samaco et al., 2013; Ward et al., 2011). Prior to PES, the propranolol cohorts and phenytoin cohorts were put through a battery of behavioral assays, weekly weights were acquired and whole-body plethysmography was performed. Additionally, heart and gonadal fat weights were collected at the end of the study.

**Fig. 3.**
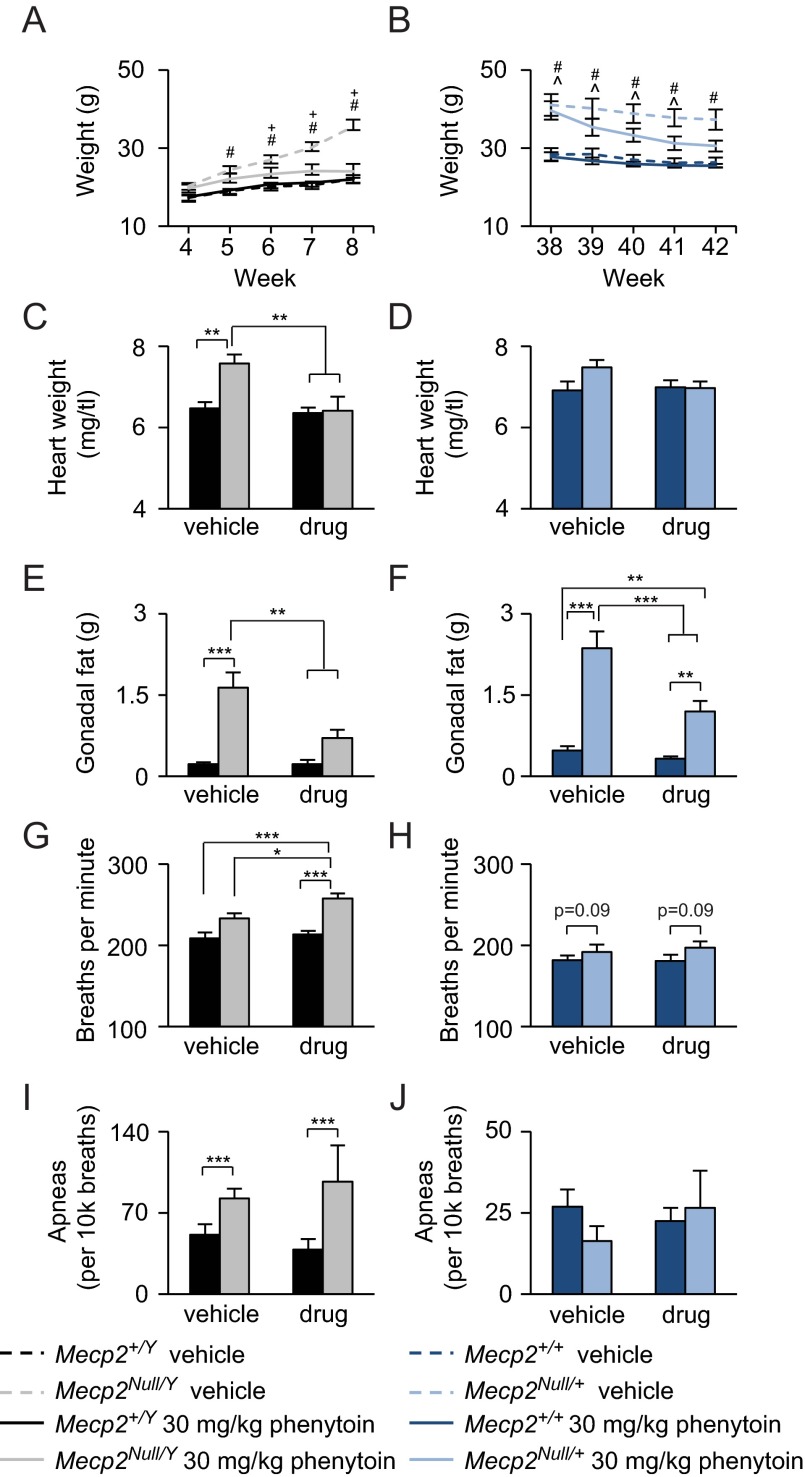
**Chronic phenytoin rescues the obesity phenotype but worsens basal hyperventilation in RTT mice.** (A,B) Phenytoin treatment rescues the obesity phenotype observed in *Mecp2^Null/Y^* and *Mecp2^Null/+^* mice. (C,D) Phenytoin treatment rescues the increased heart weight phenotype in *Mecp2^Null/Y^* mice, but did not affect the normal heart weight observed in *Mecp2^Null/+^* mice. (E,F) Phenytoin treatment decreased gonadal fat accumulation in *Mecp2^Null/Y^* and *Mecp2^Null/+^* mice. (G,H) Chronic phenytoin worsens the increased basal breathing in *Mecp2^Null/Y^* but not *Mecp2^Null/+^* mice. (I,J) Phenytoin treatment does not affect apneic events in RTT mice. Data are expressed as means±s.e.m. *n*=7–11 per genotype per treatment. *P<0.05, **P<0.01, ***P≤0.001; ^#^*P*<0.05 for *Mecp2^Null/Y^* or *Mecp2^Null/+^* vehicle versus WT vehicle or WT phenytoin; ^*P*<0.05 for *Mecp2^Null/+^* phenytoin versus WT vehicle or WT phenytoin; ^+^*P*<0.05 for *Mecp2^Null/Y^* vehicle versus *Mecp2^Null/Y^* phenytoin.

As previously reported (Pitcher et al., 2013; Ward et al., 2011), *Mecp2^Null/Y^* mice in this genetic strain background exhibited obesity, which was rescued with phenytoin treatment (Fig. 3A). These effects were observed starting at 6 weeks of age and their weights continue to improve with continued treatment (Fig. 3A). These beneficial weight loss effects were also observed in *Mecp2^Null/+^* phenytoin-treated mice (Fig. 3B). *Mecp2^Null/Y^* mice have an increased heart weight normalized to tibia length, which was rescued to wild-type levels with phenytoin treatment (Fig. 3C). Interestingly, the increased heart weight was only observed in the *Mecp2^Null/Y^* mice and not *Mecp2^Null/+^* mice (Fig. 3D). *Mecp2^Null/Y^* and *Mecp2^Null/+^* mice treated with phenytoin also had a decrease in gonadal fat accumulation (Fig. 3E,F). Finally, the breathing rate was assessed using whole-body plethysmography. The basal breathing rate of *Mecp2^Null/Y^* male mice was exacerbated with phenytoin treatment, but did not affect apneic events (Fig. 3G,I). However, these adverse effects on basal breathing rate were not observed in *Mecp2^Null/+^* phenytoin-treated mice (Fig. 3H,J).

**Fig. 4.**
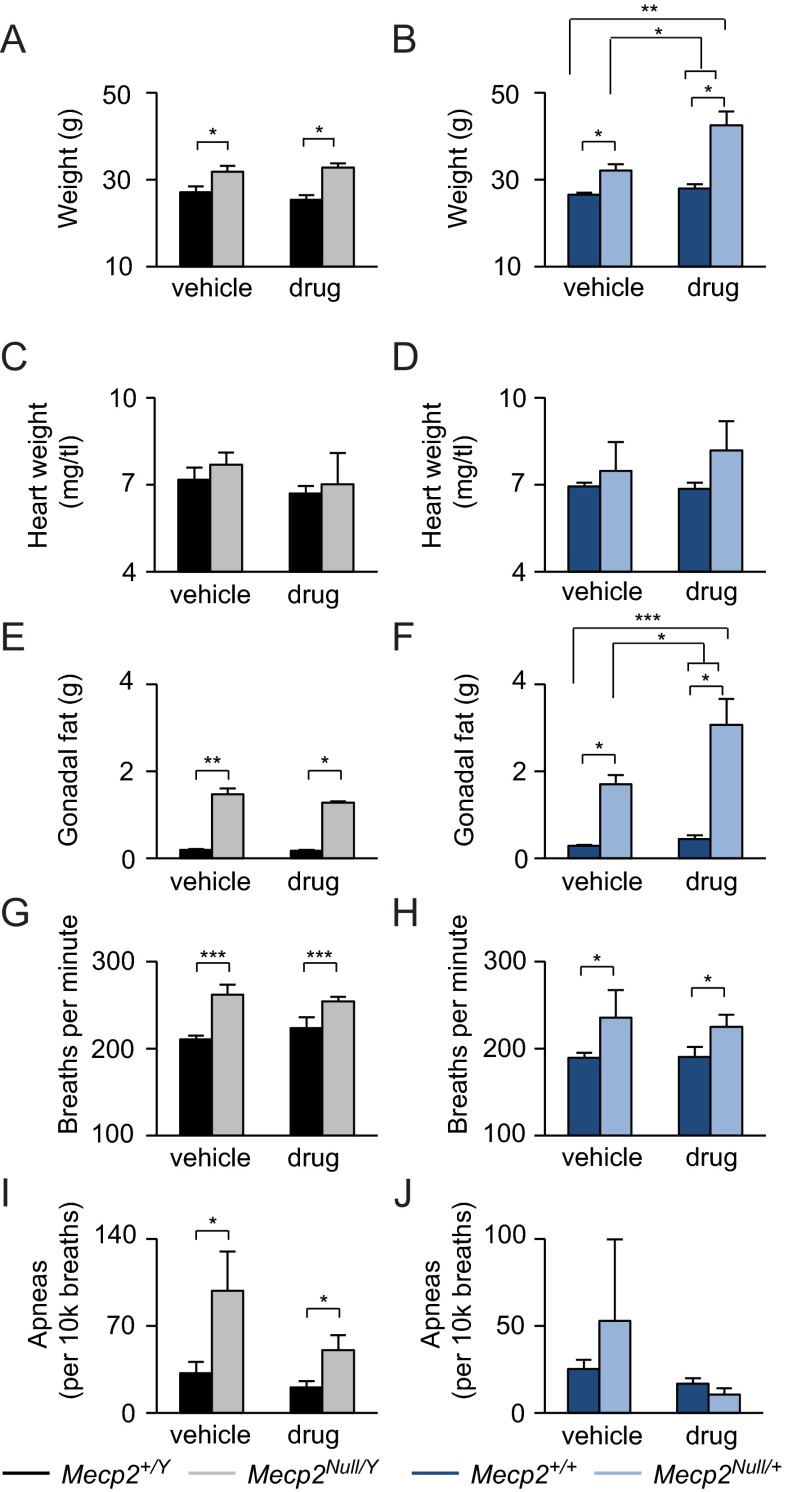
**Chronic propranolol worsens the obesity phenotype in female *Mecp2^Null/+^* mice.** (A,B) Propranolol treatment had no effect in male mice but caused an increase in body weight in *Mecp2^Null/+^* mice. (C,D) Propranolol treatment had no effect on heart weight. (E,F) Propranolol treatment increased gonadal fat accumulation in *Mecp2^Null/+^* but not *Mecp2^Null/Y^* mice. (G–J) Propranolol treatment did not affect basal breathing or apneas. Data are expressed as means±s.e.m. *n*=7–11 per genotype per treatment. *P<0.05, **P<0.01, ***P≤0.001.

Propranolol did not affect the weight of *Mecp2^Null/Y^* but did worsen the overweight phenotype in *Mecp2^Null/+^* mice (Fig. 4A,B). No effects were observed on heart weights (Fig. 4C,D). Propranolol had no effect on gonadal fat accumulation in *Mecp2^Null/Y^* mice (Fig. 4E), whereas *Mecp2^Null/+^* treated with propranolol had an increase in gonadal fat accumulation (Fig. 4F). Propranolol treatment did not affect basal breathing or apneas in RTT mice (Fig. 4G–J).

**Fig. 5.**
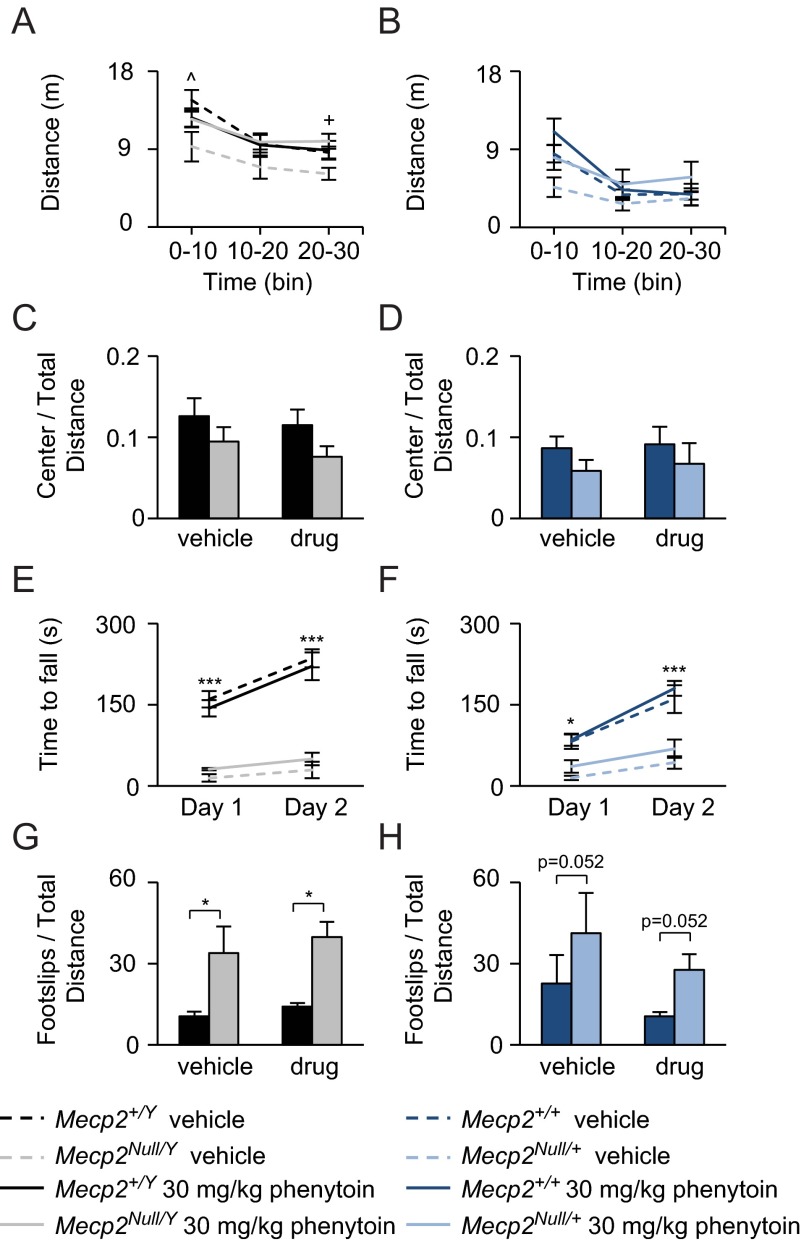
**Chronic phenytoin increases activity levels in *Mecp2^Null/Y^* mice.** (A,B) Phenytoin treatment in *Mecp2^Null/Y^* mice caused increased activity levels in the open field but had no effect on *Mecp2^Null/+^* mice. (C,D) No effects were observed on anxiety. (E,F) Motor learning was not improved with phenytoin treatment. (G,H) *Mecp2^Null/Y^* and *Mecp2^Null/+^* mice had poor motor coordination as determined by an increase in foot slips in parallel rod. Data are expressed as means±s.e.m. *n*=6–11 per genotype per treatment unless otherwise stated. *P<0.05, ***P≤0.001; ^*P*<0.05 between genotypes; ^+^*P*<0.05 for *Mecp2^Null/Y^* vehicle versus *Mecp2^Null/Y^* treated with phenytoin.

Activity and motor learning assays were performed on phenytoin-treated mice. Surprisingly, *Mecp2^Null/Y^* mice treated with phenytoin were more active in the open field assay when compared to *Mecp2^Null/Y^* vehicle-treated mice (Fig. 5A), which was not observed in *Mecp2^Null/+^* phenytoin-treated mice (Fig. 5B). No effects were observed on anxiety-related behavior (Fig. 5C,D). *Mecp2^Null/Y^* and *Mecp2^Null/+^* mice had poor performance on the parallel rod and accelerating rotating rod when compared to wild-type mice and there were no beneficial treatment effects observed on these behavioral assays (Fig. 5E–H).

### Na^+^-channel-blocking AEDs improve QTc status in people with RTT

In order to assess whether initiation of drugs that block Na^+^ channels can affect QTc intervals in people with RTT, we utilized data from the Rett Syndrome Natural History Study, which has been collecting clinical data on a cohort of people with RTT for over 10 years. Owing to increasing awareness of the increased incidence of LQT in RTT, we found ECG data on 667 people enrolled in this study. Of these, 331 had more than one ECG assessed and we focused attention on these. We determined that of the 331 with multiple ECG assessments, 68 had initiated either β-blockers or Na^+^ channel blocker AED therapies between the two ECG assessments (Table 1). Individuals on both β-blocking agents and AEDs were excluded from the analysis. We decided to look at the role of Na^+^-channel-blocking AEDs because epilepsy is common in RTT and many people with RTT are treated for seizures with these types of AEDs.

**Table 1.**
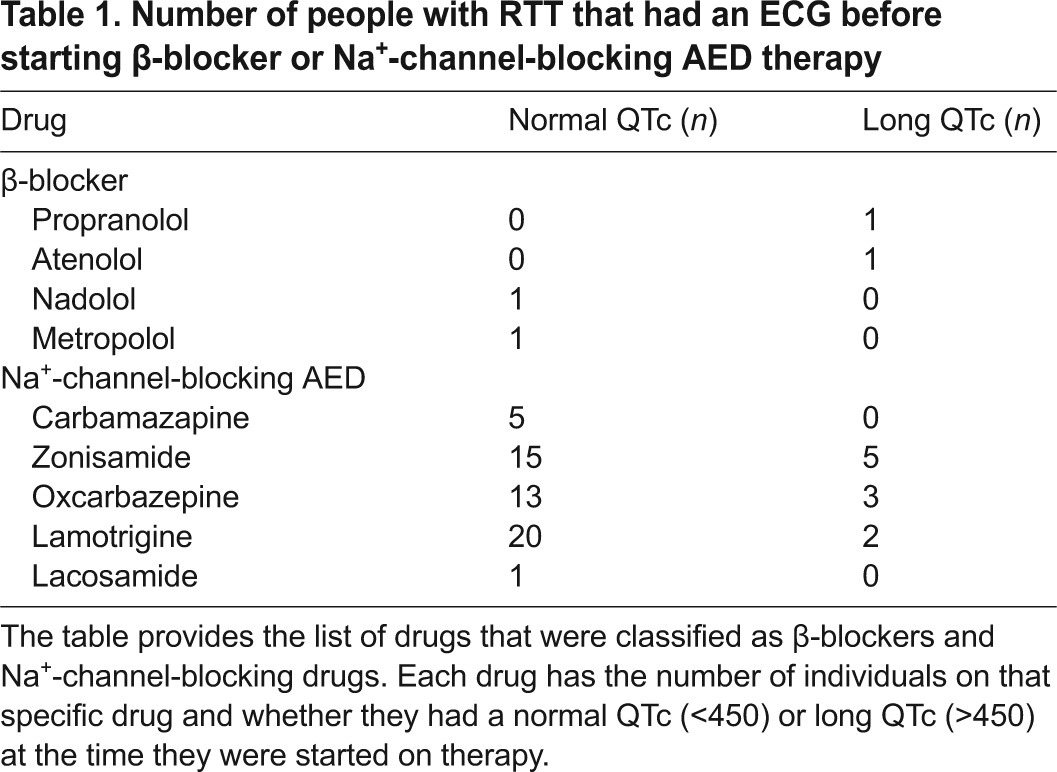
Number of people with RTT that had an ECG before starting β-blocker or Na^+^-channel-blocking AED therapy

Of these 68 individuals with multiple ECGs and specific drug initiation, only four had been started on β-blocking agents. Two had prolonged QTc (QTc >450 ms) and two had a normal QTc interval prior to drug initiation. These small numbers preclude any analysis or meaningful interpretation of the effects of β-blocking agents on the ECG.

**Fig. 6.**
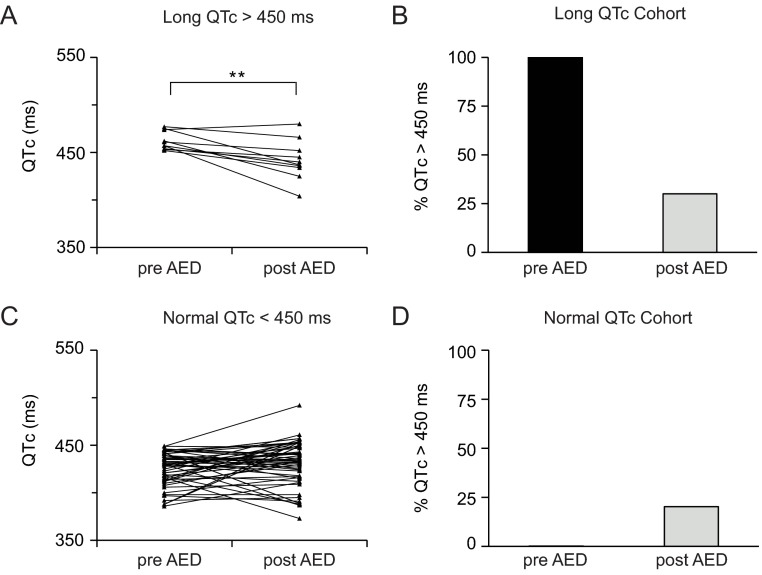
**Anti-epileptic drugs with Na^+^ channel blocking activity improve QTc in individuals with Rett syndrome.** (A) Individuals with RTT that have a QTc >450 ms show significant improvement on QTc status after AED therapy was initiated. (B) Treatment with AEDs decreased the QTc status of 70% of the individuals that have LQT below the 450 ms LQT threshold. (C) There was no significant effect on QTc status post AED therapy in individuals with a normal QTc <450 ms, but (D) there was an increase in QTc in 20.3% of the individuals. Prolonged QTc >450 ms with AEDs, *n*=10; normal QTc <450 ms with AEDs, *n*=54. **P=0.005 (paired Student’s *t*-test).

By contrast, 64 individuals with multiple ECGs had started Na^+^-channel-blocking AEDs between successive ECG assessments. Within this cohort, 54 individuals had a normal QTc interval before starting the drug, and 10 individuals had a prolonged QTc (LQT) before starting the drug. Surprisingly, individuals that had LQT prior to the start of Na^+^-channel-blocking AEDs showed a significant improvement on their QTc status on the following ECG after therapy was initiated (Fig. 6A). Additionally, 7 of the 10 individuals (70%) were below the 450 ms LQT threshold post AED therapy (Fig. 6B). There was no significant effect post AED therapy on the normal QTc group (Fig. 6C), but for 20.3% (11 of 54) the QTc was prolonged beyond the threshold for QTc prolongation after treatment (Fig. 6D).

## DISCUSSION

Sudden and unexpected death causes a substantial fraction of the mortality in RTT and the most likely proximate cause is due to cardiac arrhythmias secondary to the increased incidence of LQT syndrome. We previously found that LQT in RTT is related to an increase in persistent Na^+^ current in cardiomyocytes, and in this work we use a mouse model of RTT to demonstrate that the optimal treatment of LQT and cardiac arrhythmias is chronic Na^+^ channel blockade. Additionally, we found that the standard of care, treatment with an adrenergic β-blocking agent, is ineffective in treating LQT syndrome or preventing cardiac arrhythmias in RTT. Retrospective analysis of human natural history data supports these findings in mice, showing that the addition of a Na^+^-channel-blocking AED leads to reduction in QTc prolongation. This work indicates that the approach to treatment of LQT intervals in RTT patients needs to be re-evaluated and explored with prospective clinical trials.

Interestingly, although the primary focus of this preclinical treatment trial was on the treatment effects on cardiac arrhythmias, we noted additional treatment effects on weight, behavior and breathing. Chronic phenytoin treatment improved activity in male RTT animals and improved weight in both male and female RTT animals. In contrast, chronic propranolol worsened the obesity seen in female RTT mice. The beneficial effects of phenytoin on activity were unexpected. It is possible that phenytoin treatment decreased seizure activity in these animals, leading to increased activity; however, no obvious seizures were observed in any groups. The increased activity could be a factor in the decreased obesity observed; however, the improvement in weight was seen in both genders whereas the increased activity was only observed in male animals. It might be that there are central nervous system (CNS) abnormalities leading to obesity that respond favorably to Na^+^ channel blockade, such as increased persistent Na^+^ current within the CNS neurons. This is an avenue for future work.

In contrast to the beneficial effects of chronic phenytoin treatment observed on weight and activity, this treatment worsened basal hyperventilation in male *Mecp2^Null/Y^* mice. Interestingly, chronic treatment with phenytoin can alter monoamine levels (Meshkibaf et al., 1995), which can disrupt the respiratory network. Furthermore, the abnormal breathing patterns in *Mecp2^Null/Y^* can be attributed to the age-dependent decrease in monoamine levels (Panayotis et al., 2011; Samaco et al., 2009). Thus, phenytoin might be worsening the basal hyperventilation by causing a further reduction in monoamines in the RTT mice. This is a potentially concerning effect, but fortunately this was not observed for apneas in male *Mecp2^Null/Y^* mice, and there was no effect of phenytoin on breathing on female *Mecp2^Null/+^* mice, the most appropriate model of human disease. Nonetheless, this effect on breathing is a feature that needs to be explored in greater depth both in the animal model as well as in people with RTT.

Notably, in our study *Mecp2^Null/Y^* mice treated with phenytoin showed an increase in activity and hyperventilation, whereas these effects were not observed in the less-severe female *Mecp2^Null/+^* mice. However, both male *Mecp2^Null/Y^* and female *Mecp2^Null/+^* mice treated with phenytoin showed an improvement in their weight and the cardiac phenotypes. Although RTT is almost exclusively studied using the male *Mecp2^Null/Y^* mouse model, owing to the consistent early onset of the phenotypes when compared to the more heterogeneous onset of the phenotypes in the female *Mecp2^Null/+^* mouse model, our study suggests that both models should be investigated when designing pre-clinical experiments.

Phenytoin was chosen for the preclinical treatment trial as a representative Na^+^ channel blocking AED; however, phenytoin has many untoward properties, including distinct detrimental effects on cardiac function that can lead to atrioventricular block (Leonard, 1958; Randazzo et al., 1995; Scherf et al., 1960), that make this a suboptimal choice for treatment owing to the incidence of sinoatrial block and atrioventricular block in people with RTT (Guideri and Acampa, 2005; Madan et al., 2004). A number of AEDs block Na^+^ channels: our retrospective analysis suggests that these agents might be effective in treating LQT and preventing sudden cardiac death in RTT. Because of the high incidence of seizures in RTT, choosing an agent that targets both CNS issues (seizures) as well as cardiac issues (LQT) might prove advantageous. However, compounds that specifically target the persistent Na^+^ current, such as ranolazine, might be the most effective agent (Huang et al., 2011; Kahlig et al., 2010; Shah et al., 2012). Future work will focus on using the cellular and animal models of RTT to identify the optimal agent to move into human clinical trials.

One limitation of this study is the retrospective nature of the human data, which limits interpretation. This is most apparent for the analysis of individuals started on β-blockers. Only two individuals with LQT were exclusively on β-blockers, thus we could not make any conclusions regarding the effect of β-blockers on LQT in people with RTT. Further prospective studies should be implemented directly comparing individuals with RTT and LQT randomly assigned to β-blocker therapy or Na^+^ channel blocker therapy.

There are a number of other neurological conditions that also show cardiac rhythm abnormalities. For example, people with epilepsy are at risk of Sudden Death in Epilepsy (SUDEP), which is believed to be caused by cardiac problems mediated by neuronal dysfunction (Massey et al., 2014). Seizures have also been reported to cause ventricular fibrillation (Ferlisi et al., 2013) and prolonged QT (Brotherstone et al., 2010). In animal models, repetitive seizures can induce remodeling of the Na^+^ and K^+^ channels within the heart (Bealer et al., 2010). One specific epilepsy disorder that has increased risk of sudden death, Dravet Syndrome, is caused by mutations in the Na^+^ channel SCN1A, which is expressed both in the heart and the brain (Gong et al., 1999; Maier et al., 2002; Maier et al., 2003; Westenbroek et al., 1989). Recent work in mice has demonstrated that loss of SCN1A solely within the nervous system leads to a surge of the parasympathetic nervous system during seizure activity causing lethal bradycardia, whereas no cardiac abnormalities or death is observed when SCN1A is removed solely from the heart (Kalume et al., 2013). Additionally, mouse models of RTT have previously been reported to have seizure-like events (Colic et al., 2013; Ward et al., 2011). Whether these seizure-like events correlate with cardiac abnormalities has not been investigated. Notably, seizures can cause sudden death due to an imbalance in the autonomic nervous system (i.e. sympathetic versus parasympathetic). Individuals with RTT also present with autonomic abnormalities (Julu et al., 1997), which raises the question of whether autonomic nervous system imbalance might be causing the cardiac abnormalities. Interestingly, Na^+^ channel blockers, such as phenytoin and carbamazepine, can have direct and indirect effects on cardiac autonomic modulation (Kennebäck et al., 1997), which raises the question of whether phenytoin might be normalizing these autonomic imbalances, thus preventing arrhythmias in our model of RTT. Further work using animal models of RTT will allow for a more in-depth investigation into the mechanism of how neuronal abnormalities can cause LQT and arrhythmias in mouse models of RTT, which might lead to novel therapeutics for the management of LQT in RTT.

## MATERIALS AND METHODS

### Study approval for human subjects

The Rett Syndrome Natural History Study (https://clinicaltrials.gov, identified NCT00296764) is a longitudinal study of the clinical features of RTT and was approved by the institutional review boards of the participating centers (Baylor College of Medicine, University of Alabama at Birmingham, Greenwood Genetics Center, and Boston Children’s Hospital). Written informed consent was obtained prior to inclusion in this study. Data were collected as previously described (Glaze et al., 2010; Neul et al., 2014). For this work, we retrospectively analyzed ECG data collected from 667 individuals. Data was first filtered to obtain only QTc values from individuals that had an ECG taken prior to and after the start of either β-blockers or Na^+^-channel-blocking AED therapies (Table 1). Data were then filtered to remove any individuals that were on both β-blockers and AEDs. Finally, we further divided the data into cohorts of individuals with a prolonged QTc≥450 ms, as previously defined (McCauley et al., 2011) (β-blockers *n*=2 and AEDs *n*=10), and individuals with a normal QTc<450 ms (β-blockers *n*=2 and AEDs *n*=54) (Table 1).

### Animals

All research and animal care was approved by the Baylor College of Medicine Institutional Animal Care and Use Committee and animals were housed in AAALAC approved facilities. Experimental animals were generated by mating *Mecp2^Null/+^* (Mecp2^TM1.1Bird^, JAX #003890) female in a 129S6 background to a C57Bl6 male mouse. Heterozygous and hemizygous mutant and wild-type isogenic B6129S6F1 mice were used for the following experiments.

### Experimental design

Male (*Mecp2^Null/Y^*), female (*Mecp2^Null/+^*), and littermate wild-type (WT) controls were randomized and assigned to a treatment cohort. Mice were treated with either 30 mg/kg body weight of phenytoin twice a day or 10 mg/kg body weight of propranolol administered via osmotic pump. The experimenter was blinded to treatment and genotype. Mice were assessed for changes in ECG interval parameters, incidence of sustained ventricular tachycardias, behavioral and physiological phenotypes.

### Drug treatments

Mice were randomly assigned to a treatment group. The experimenter was blinded to treatment (vehicle or drug) as well as the genotype of the mice throughout the experimental process of obtaining behavioral and physiological data. *Mecp2^Null/Y^* and wild-type control treatments began at 4 weeks of age and were treated for 28 days. Meanwhile, *Mecp2^Null/+^* mice and wild-type control mice started treatment at 9 months for 28 days. The phenytoin cohort was administered 30 mg/kg body weight of phenytoin dissolved in 1% Tween 80 or vehicle two times a day via intraperitoneal injection. Propranolol was dissolved in 0.9% NaCl with 2% ascorbic acid and administered subcutaneously via osmotic pump (Alzet model 2004) at a rate of 0.25 μl/hour for a dose of 10 mg/kg body weight/day. These doses were chosen as standard effective doses that achieve high serum levels in mice (Applegate et al., 1997; Naghshin et al., 2009).

### Surface ECG

Surface ECGs were taken before programmed electrical stimulation (PES) was performed. Mice were anesthetized with 1.5% isoflurane in 95% O_2_ and six-lead ECGs were recorded as previously outlined (Li and Wehrens, 2010) by pad electrodes with band-pass filtering between 0.03 Hz and 1 kHz. The mean of ten interval measurements per mouse per genotype were used for the PR, QRS, QT and RR interval final values. Corrected QT intervals (QTc) were calculated by the formula QTc = QT + 0.3173 × (170 − RR) (Nuyens et al., 2001).

### Programmed electrical stimulation

Atrial and ventricular intracardiac electrograms were recorded with a 1.1-F octapolar electrode catheter that was carefully placed into the right ventricle via the right jugular vein exactly as previously described (Li and Wehrens, 2010). Programmed electrical stimulus of overdrive pacing and extra stimulus protocols were used to test the susceptibility to VT. Sustained VT is defined as VT lasting more than 1 s (McCauley et al., 2011).

### Behavior assays

All behavior phenotyping was performed after 2–3 weeks of treatment. The behavior battery was performed on *Mecp2^Null/Y^* mice between 7 and 8 weeks. *Mecp2^Null/+^* mice were tested at 38–39 weeks. Behavior assessments of open field, accelerating rotating rod and parallel rod walking were performed as previously described (Samaco et al., 2008).

#### Open-field analysis

Mice were placed in a room with 60 dB white noise and 60 lux illumination. They were placed in a chamber and activity was recorded using photobeams connected to the computer-operated Digiscan optical animal activity system. Mice were left in the chamber for 30 minutes.

#### Accelerating rotating rod

Mice were placed on a rotating rod for a maximum time of 5 minutes. The time to fall was recorded for each mouse. The minimum speed was 4.0 rpm, and the maximum speed was 40 rpm. Mice were tested four times for 2 days (total of eight trials). There was a 30-minute wait between each trial. The mean time to fall was calculated for each day.

#### Parallel rod walking

Parallel rod walking was performed as previously described (Pitcher et al., 2013). Mice were placed in a chamber containing parallel metal bars spaced 8 cm apart and suspended 1 cm above a metal floor. A circuit was complete when a foot touched the metal floor, which was considered as a foot slip. Locomotor activity was recorded using ANY-maze software synced to an overhead camera. Distance traveled was used to normalize the number of foot slips. Mice were allowed to walk on the parallel rods for 10 minutes.

### Physiological recordings

#### Telemetry

Mice were implanted with a DSI ETA F-10 telemeter (Data Sciences International, St Paul, MN) between 3–4 weeks of age, as previously reported (McCauley and Wehrens, 2010). ECG output was recorded by using a receiver matrix coupled to data acquisition software program (Ponemah; Data Sciences International, St Paul, MN). Data was filtered for artifacts to obtain accurate RR interval calls by enabling ECG Pro’s noise detection filter.

#### Plethysmography

Plethysmography was performed as previously reported (Ward et al., 2011). Briefly, mice were placed in an unrestrained whole-body plethysmography chamber and were allowed to habituate for 20 minutes followed by a 30-minute baseline recording. Data was acquired using the Ponemah Software which was then exported for analysis using MATLAB where the data was then filtered for movement artifacts.

### Statistics

Two-way ANOVA for genotype and treatment effects followed by a one-way ANOVA for multiple comparisons was used to determine statistical significances. Repeated measure ANOVA was used for weekly weight analysis. Categorical variables were compared using Pearson Chi-Square followed by Fisher’s exact test for comparisons between pairs of data. Paired Student’s *t*-tests were used where appropriate. *P*<0.05 was considered significant. All analyses were performed using SPSS version 20 (SPSS, Chicago, IL).

## Supplementary Material

Supplementary Material
