# Supplementary material for: Treatment of cardiac arrhythmias in a mouse model of Rett syndrome with Na+-channel-blocking antiepileptic drugs
Source: Dis Model Mech. 2015 Feb 20;8(4):363–71. doi: 10.1242/dmm.020131 (PMC4381335; doi:10.1242/dmm.020131)
Supplement: Supplementary Material [file supp_8_4_363__index.html]

Treatment of cardiac arrhythmias in a mouse model of Rett syndrome with Na+-channel-blocking antiepileptic drugs — Supplementary Material 

# Treatment of cardiac arrhythmias in a mouse model of Rett syndrome with Na+-channel-blocking antiepileptic drugs

## DMM020131 Supplementary Material

**Files in this Data Supplement:**

- **Supplementary Material**
