# Supplementary material for: Treatment of cardiac arrhythmias in a mouse model of Rett syndrome with Na+-channel-blocking antiepileptic drugs
Source: Dis Model Mech. 2015 Feb 20;8(4):363–71. doi: 10.1242/dmm.020131 (PMC4381335; doi:10.1242/dmm.020131)
Supplement: Supplementary Material [file supp_8.4.363_DMM020131.pdf]

Supplemental material

Table S1. Electrophysiological intervals and cardiac refractory periods from wild-type (WT) vehicle, wild-type propranolol, *Mecp2*$^{Null/Y}$ vehicle, and *Mecp2*$^{Null/Y}$ propranolol treated mice at 2 months old. Data are expressed as means ± SEM. RR, interval between two consecutive R waves; PQ, interval from the start of the P wave to the peak of the Q wave; QRS, interval from the start of the Q wave to the peak of the S wave; QT, duration from the beginning of the Q wave to the end of the T wave; QTc, corrected QT interval for heart rate; SNRT. sinoatrial node refractory time defined as the last atrial pace to the first A wave; AVERP, time between pacing stimulus when atrioventricular node does not conduct; VERP, time between pacing cycle that causes ventricular refractory period. Statistical analysis was conducted for genotype and treatment effects followed by a one way ANOVA for multiple comparisons.

	**Vehicle**		**Propranolol**	
	Mecp2$^{+/Y}$ **(2 months) (n=8)**	***Mecp2***$^{Null/Y}$ **(2 months) (n=8)**	***Mecp2***$^{+/Y}$ **(2 months) (n=8)**	***Mecp2***$^{Null/Y}$ **(2 months) (n=8)**
**RR (ms)**	123.0 ± 5.9	115.2 ± 4.0	127.7 ± 6.3	119.5 ± 2.0
**PQ (ms)**	35.3 ± 0.4	34.6 ± 0.6	35.9 ± 0.9	35.2 ± 0.7
**QRS (ms)**	8.5 ± 0.1	9.4 ± 0.3$^{ł\,*}$	8.5 ± 0.2	10.3 ± 0.2$^{ł\,***}$
**QT (ms)**	33.2 ± 1.4	43.1 ± 1.3$^{ł\,**}$	35.2 ± 2.0	42.5 ± 1.0$^{ł\,*}$
**QTc (ms)**	48.1 ± 1.3	60.5 ± 0.8$^{ł\,***}$	48.6 ± 1.8	58.5 ± 1.0$^{ł\,***}$
**SNRT (ms)**	142.9 ± 9.7	132.0 ± 7.2	167.8 ± 11$^{\delta}$	162.0 ± 12$^{\delta}$
**AVERP (ms)**	44.8 ± 1.5	42.4 ± 1.4	46.0 ± 2.2	45.2 ± 1.9
**VERP (ms)**	23.3 ± 1.2	23.6 ± 1.7	23.8 ± 1.6	23.1 ± 1.0

Genotype effect: WT vs *Mecp2*$^{Null/Y}$ ł $p<0.001$; Treatment effect: vehicle vs propranolol $^{\delta}$ $p<0.05$; ANOVA Tukey's post hoc *Mecp2*$^{Null/Y}$ vehicle or propranolol vs WT vehicle or propranolol * $p<0.05$, ** $p<0.01$, *** $p<0.001$

Table S2. Electrophysiological intervals and cardiac refractory periods from wild-type (WT) vehicle, wild-type propranolol, *Mecp2*$^{Null/+}$ vehicle, and *Mecp2*$^{Null/+}$ propranolol treated mice at 10 months old. Data are expressed as means ± SEM.

	**Vehicle**		**Propranolol**	
	Mecp2$^{+/+}$ **(10 months) (n=9)**	***Mecp2***$^{Null/+}$ **(10 months) (n=9)**	***Mecp2***$^{+/+}$ **(10 months) (n=8)**	***Mecp2***$^{Null/+}$ **(10 months) (n=9)**
**RR (ms)**	124.4 ± 2.7	128.4 ± 3.0	138.4 ± 4.7$^{\delta}$	129.4 ± 2.9$^{\delta}$
**PQ (ms)**	35.7 ± 0.6	36.9 ± 0.7	36.9 ± 0.8	37.8 ± 1.2
**QRS (ms)**	8.1 ± 0.1	9.9 ± 0.2^{ɨ***}	8.4 ± 0.2	10.0 ± 0.1^{ɨ***}
**QT (ms)**	33.4 ± 1.1	39.1 ± 1.2^{ʕ}	38.4 ± 1.9$^{\delta *}$	40.3 ± 1.1$^{\delta ʕ *}$
**QTc (ms)**	47.9 ± 0.6	52.3 ± 0.7^{ɨ**}	48.4 ± 0.8	53.2 ± 0.8^{ɨ**}
**SNRT**	145.1 ± 7.7	129.0 ± 4.3	182.1 ± 12$^{\frown\delta}$	155.1 ± 9.0$^{\frown\delta}$
**AVERP**	44.6 ± 0.8	44.4 ± 1.0	47.4 ± 1.6	47.0 ± 2.0
**VERP**	22.8 ± 1.1	22.4 ± 1.1	23.8 ± 1.2	24.2 ± 1.8

Genotype effect: WT vs *Mecp2*$^{Null/Y}$ ɨ p<0.001, ʕ p<0.01 ͡ p<0.05; Treatment effect: vehicle vs propranolol $^{\delta}$ p<0.05 ANOVA Tukey's post hoc *Mecp2*$^{Null/Y}$ vehicle or propranolol vs WT vehicle or propranolol * p<0.05, ** p<0.01, *** p<0.001

Table S3. Electrophysiological intervals and cardiac refractory periods from wild-type (WT) vehicle, wild-type phenytoin, *Mecp2*$^{Null/Y}$ vehicle, and *Mecp2*$^{Null/Y}$ phenytoin treated mice at 2 months old. Data are expressed as means ± SEM.

	**Vehicle**		**Phenytoin**	
	Mecp2$^{+/Y}$ **(2 months) (n=9)**	***Mecp2***$^{Null/Y}$ **(2 months) (n=7)**	***Mecp2***$^{+/Y}$ **(2 months) (n=8)**	***Mecp2***$^{Null/Y}$ **(2 months) (n=8)**
**RR (ms)**	119.3 ± 2.9	113.9 ± 4.2	115.4 ± 3.8	122.2 ± 5.3
**PQ (ms)**	35.0 ± 0.3	35.4 ± 0.3	33.3 ± 0.6	33.5 ± 0.8
**QRS (ms)**	8.3 ± 0.2	9.9 ± 0.2***	8.5 ± 0.1	7.9 ± 0.2
**QT (ms)**	31.4 ± 1.3	42.0 ± 0.9**	32.2 ± 1.4	36.0 ± 2.5
**QTc (ms)**	47.5 ± 0.8	60.0 ± 0.8***	49.6 ± 1.1	51.2 ± 1.3
**SNRT**	134.6 ± 4.8	140.1 ± 11	156.8 ± 11	140.0 ± 9.5
**AVERP**	45.5 ± 1.1	43.5 ± 3.7	44.5 ± 1.6	47.6 ± 2.3
**VERP**	23.7 ± 1.2	24.7 ± 1.4	24.0 ± 1.1	23.8 ± 1.8

ANOVA Tukey's post hoc QRS and QTc *Mecp2*$^{Null/Y}$ vehicle vs *Mecp2*$^{Null/Y}$ phenytoin, WT vehicle, or WT phenytoin*** p<0.001; QT *Mecp2*$^{Null/Y}$ vehicle vs WT vehicle or WT phenytoin*** p<0.001

Table S4. Electrophysiological intervals and cardiac refractory periods from wild-type (WT) vehicle, wild-type phenytoin, *Mecp2*$^{Null/+}$ vehicle, and *Mecp2*$^{Null/+}$ phenytoin treated mice at 10 months old. Data are expressed as means ± SEM.

	**Vehicle**		**Phenytoin**	
	Mecp2$^{+/+}$ **(10 months) (n=10)**	***Mecp2***$^{Null/+}$ **(10 months) (n=9)**	***Mecp2***$^{+/+}$ **(10 months) (n=9)**	***Mecp2***$^{Null/+}$ **(10 months) (n=9)**
**RR (ms)**	119.0 ± 3.3	115.3 ± 2.5	115.4 ± 2.6	123.6 ± 3.6
**PQ (ms)**	35.9 ± 0.5	34.0 ± 0.6	35.2 ± 0.7	36.9 ± 0.7*
**QRS (ms)**	7.9 ± 0.1	9.9 ± 0.1***	8.0 ± 0.1	8.1 ± 0.1
**QT (ms)**	32.8 ± 1.2	37.6 ± 0.7**	31.6 ± 0.8	35.1 ± 1.2
**QTc (ms)**	49.0 ± 0.2	55.0 ± 0.8***	48.9 ± 0.4	49.8 ± 0.4
**SNRT**	138.8 ± 5.8	153.1 ± 8.7**	128.9 ± 4.1	166.1 ± 7.8
**AVERP**	45.1 ± 0.8	45.4 ± 1.5*	42.0 ± 1.7	50.6 ± 1.4
**VERP**	22.3 ± 1.1	24.4 ± 1.5	25.0 ± 1.1	27.2 ± 0.8

ANOVA Tukey's post hoc PQ: *Mecp2*$^{Null/+}$ vehicle vs *Mecp2*$^{Null/+}$ phenytoin * p<0.05; QRS and QTc: *Mecp2*$^{Null/+}$ vehicle vs *Mecp2*$^{Null/+}$ phenytoin, WT vehicle, or WT phenytoin*** p<0.001; QT: *Mecp2*$^{Null/Y}$ vehicle vs WT vehicle or WT phenytoin** p<0.01; SNRT: *Mecp2*$^{Null/+}$ vehicle vs *Mecp2*$^{Null/+}$ phenytoin **p<0.01; AVERP: *Mecp2*$^{Null/+}$ vehicle vs *Mecp2*$^{Null/+}$ phenytoin *p<0.05
